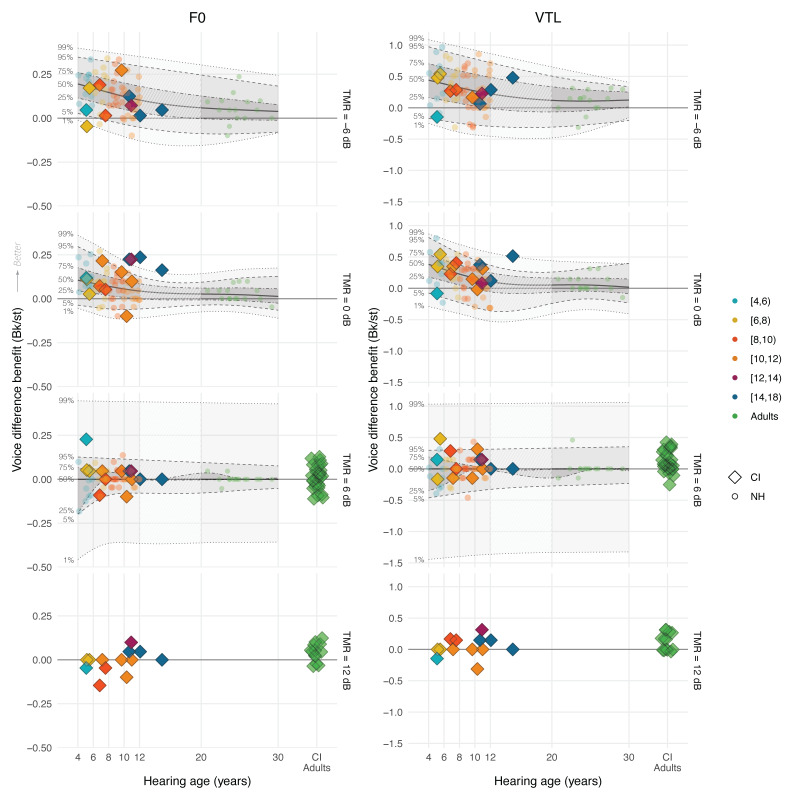# Prelingually Deaf Children With Cochlear Implants Show Better Perception of Voice Cues and Speech in Competing Speech Than Postlingually Deaf Adults With Cochlear Implants

**DOI:** 10.1097/AUD.0000000000001489

**Published:** 2024-04-15

**Authors:** Leanne Nagels, Etienne Gaudrain, Deborah Vickers, Petra Hendriks, Deniz Başkent

**Affiliations:** 1 Center for Language and Cognition Groningen (CLCG), University of Groningen, Groningen, The Netherlands; 2 Department of Otorhinolaryngology/Head and Neck Surgery, University Medical Center Groningen, University of Groningen, Groningen, The Netherlands; 3 Research School of Behavioural and Cognitive Neurosciences, University of Groningen, Groningen, The Netherlands; 4 CNRS UMR 5292, Lyon Neuroscience Research Center, Auditory Cognition and Psychoacoustics, Inserm UMRS 1028, Université Claude Bernard Lyon 1, Université de Lyon, Lyon, France; 5 Cambridge Hearing Group, Sound Lab, Clinical Neurosciences Department, University of Cambridge, Cambridge, United Kingdom; 6 W.J. Kolff Institute for Biomedical Engineering and Materials Science, University Medical Center Groningen, University of Groningen, Groningen, The Netherlands.

**Keywords:** Cochlear implants, Cognition, Development, Prelingual deafness, Voice perception

## Abstract

**Objectives:**

Postlingually deaf adults with cochlear implants (CIs) have difficulties with perceiving differences in speakers’ voice characteristics and benefit little from voice differences for the perception of speech in competing speech. However, not much is known yet about the perception and use of voice characteristics in prelingually deaf implanted children with CIs. Unlike CI adults, most CI children became deaf during the acquisition of language. Extensive neuroplastic changes during childhood could make CI children better at using the available acoustic cues than CI adults, or the lack of exposure to a normal acoustic speech signal could make it more difficult for them to learn which acoustic cues they should attend to. This study aimed to examine to what degree CI children can perceive voice cues and benefit from voice differences for perceiving speech in competing speech, comparing their abilities to those of normal-hearing (NH) children and CI adults.

**Design:**

CI children’s voice cue discrimination (experiment 1), voice gender categorization (experiment 2), and benefit from target-masker voice differences for perceiving speech in competing speech (experiment 3) were examined in three experiments. The main focus was on the perception of mean fundamental frequency (F0) and vocal-tract length (VTL), the primary acoustic cues related to speakers’ anatomy and perceived voice characteristics, such as voice gender.

**Results:**

CI children’s F0 and VTL discrimination thresholds indicated lower sensitivity to differences compared with their NH-age-equivalent peers, but their mean discrimination thresholds of 5.92 semitones (st) for F0 and 4.10 st for VTL indicated higher sensitivity than postlingually deaf CI adults with mean thresholds of 9.19 st for F0 and 7.19 st for VTL. Furthermore, CI children’s perceptual weighting of F0 and VTL cues for voice gender categorization closely resembled that of their NH-age-equivalent peers, in contrast with CI adults. Finally, CI children had more difficulties in perceiving speech in competing speech than their NH-age-equivalent peers, but they performed better than CI adults. Unlike CI adults, CI children showed a benefit from target-masker voice differences in F0 and VTL, similar to NH children.

**Conclusion:**

Although CI children’s F0 and VTL voice discrimination scores were overall lower than those of NH children, their weighting of F0 and VTL cues for voice gender categorization and their benefit from target-masker differences in F0 and VTL resembled that of NH children. Together, these results suggest that prelingually deaf implanted CI children can effectively utilize spectrotemporally degraded F0 and VTL cues for voice and speech perception, generally outperforming postlingually deaf CI adults in comparable tasks. These findings underscore the presence of F0 and VTL cues in the CI signal to a certain degree and suggest other factors contributing to the perception challenges faced by CI adults.

## INTRODUCTION

Speech perception in complex listening scenarios, such as understanding a talker in the presence of other interfering talkers, remains challenging for many cochlear implant (CI) users because of the limited spectrotemporal fine structure cues in CI-transmitted signals (see the review by Başkent et al. 2016). Although speakers’ voice characteristics aid normal-hearing (NH) listeners when segregating different speech streams (Cherry 1953; Bregman 1994), CI users often have difficulties in perceiving differences in voice characteristics (Gaudrain & Başkent 2018; El Boghdady et al. 2019; Meister et al. 2020), voice gender (Meister et al. 2009, 2016; Fuller et al. 2014; Skuk et al. 2020), prosody (Chatterjee & Peng 2008; Meister et al. 2009; Peng et al. 2012; Everhardt et al. 2020), or vocal emotions (Luo et al. 2007; Most & Aviner 2009; Chatterjee et al. 2015). Previous voice-perception studies have mainly focused on postlingually deaf CI adults, who became deaf after language foundations were well established, leaving limited knowledge about prelingually deaf implanted CI children with congenital or early-onset severe to profound deafness during the critical stages of language development (e.g., Manrique et al. 1999; Cleary et al. 2005; Heywood et al. 2016).

Postlingually deaf CI adults have to adapt their existing acoustic representations to use the reduced acoustic cues from the CI. In contrast, prelingually deaf CI children’s voice perception may be affected by cognitive or language abilities that improve with age, such as selective auditory attention or auditory working memory, as observed for NH children (e.g., Buss et al. 2017; Flaherty et al. 2019; Nagels et al. 2020a). Unlike postlingually deaf adult CI users, the auditory representations of prelingually deaf CI children are primarily based on the spectrotemporally degraded CI signal. Combined with the extensive neuroplastic changes during childhood (Manrique et al. 1999; Kral & Sharma 2012), this may make CI children more effective at using the degraded acoustic cues than CI adults. Alternatively, the lack of exposure to normal acoustic hearing may make their internal acoustic representations less robust than those of CI adults. Hence, this study investigated prelingually deaf CI children’s ability to perceive voice cues and benefit from voice differences for perceiving speech in competing speech. We also examined how their abilities compare to those of NH children and postlingually deaf CI adults who were tested in previous studies using similar experiments (Fuller et al. 2014; Gaudrain & Başkent 2018; El Boghdady et al. 2019, 2021; Nogueira et al. 2021). Finally, we examined the relationship between three voice-perception abilities involving different cognitive processes, ranging from voice discrimination to voice categorization and the perception of speech in competing speech.

### Perception of Voice Characteristics via CIs

Prior research on voice perception in CI users has primarily focused on fundamental frequency (F0) or its perceptual correlate “voice pitch” perception (see the review by Moore & Carlyon 2005). Speakers’ mean F0 is a primary physiologically related acoustic cue that characterizes a speaker’s voice, such as their perceived voice gender (Titze 1989; Smith & Patterson 2005; Skuk & Schweinberger 2014), and is determined by the glottal pulse rate. CI users show reduced discrimination of F0-related cues compared with NH listeners, affecting, for instance, perception of lexical tones (Peng et al. 2004; Wei et al. 2004), prosody (Chatterjee & Peng 2008; Meister et al. 2009; Peng et al. 2012; Everhardt et al. 2020), and pitch contours (Deroche et al. 2016, 2019). Gaudrain and Başkent (2018), using voice stimuli taken from naturally produced speech tokens, found that postlingually deaf CI adults had a mean just-noticeable difference (JND) in mean F0 of 9.19 st (70.0%), about 4.7 times larger than the 1.95 st (11.9%) F0 JND of NH listeners. A group difference of comparable magnitude was observed by Meister et al. (2011), while using sentences instead of nonword stimuli.

Besides mean F0 discrimination, Gaudrain and Başkent (2018) investigated postlingually deaf CI adults’ perception of vocal-tract length (VTL), which is another physiologically related and relatively unchangeable voice characteristic (Kreiman & Sidtis 2011). Speakers’ VTL is mainly determined by the distance between their vocal folds and the aperture of their oral and nasal cavities, which closely relates to speakers’ height (Fitch & Giedd 1999). Together with mean F0, speakers’ VTL predominantly defines their perceived voice gender (Titze 1989; Smith & Patterson 2005; Skuk & Schweinberger 2014). Gaudrain and Başkent (2018) found that CI adults had a mean VTL JND of 7.19 st (51.5%), which was 4.2 times larger than the mean VTL JND of 1.73 st (10.5 %) observed in NH listeners. Although F0 and VTL discrimination are both impaired in CI adults, the impact of elevated JNDs on voice gender perception is considerably larger for VTL than F0 (Fuller et al. 2014). Although many CI adults can perceive a typical voice gender difference of 9 st in mean F0, a typical 3.6 st difference in VTL is inaudible to almost all CI adults based on their discrimination thresholds.

To examine the specific contribution of mean F0 and VTL cues to voice gender categorization, the F0 and VTL parameters of the same speaker were artificially manipulated in some studies using a resynthesis procedure to keep all other voice-related cues consistent (Fuller et al. 2014; Meister et al. 2016; Skuk et al. 2020). These studies showed that CI adults use F0 cues for voice gender categorization to a similar degree as NH listeners despite having elevated F0 JNDs. However, CI adults showed a reduced weighting of VTL cues across the whole stimulus continuum, different than NH listeners, who make use of both mean F0 and VTL (Fuller et al. 2014; Meister et al. 2016; Skuk et al. 2020). Combined with the results of Gaudrain and Başkent (2018), these findings suggest that VTL cues are not entirely adequately transmitted via CIs, and CI adults cannot reliably determine speakers’ voice gender based on the CI-transmitted VTL cues. Nevertheless, CI adults’ weighting of VTL cues seems to increase when sentences instead of word stimuli are used (Meister et al. 2016).

The few studies that have examined voice perception in prelingually deaf CI children show some disparities with findings from CI adults, such as differences in their processing of spectrotemporal information (Jung et al. 2012; DiNino & Arenberg 2018; Landsberger et al. 2018). Kovačić and Balaban (2010) observed highly variable voice gender categorization abilities in prelingually deaf CI children based on mean F0 differences, inversely related to the duration of auditory deprivation before CI implantation. Zaltz et al. (2018) showed that prelingually deaf CI adults who were implanted earlier showed better VTL discrimination. Hence, VTL cues may be present in the spectrotemporally degraded CI signal, although reduced and likely distorted, but postlingually deaf CI adults may not effectively interpret these cues.

Perception of speech in competing speech seems to be a challenge for CI adults, partly because of their reduced sensitivity to voice characteristic differences, which helps NH listeners to segregate target from masker speech (Brungart 2001; Brungart et al. 2001; Schneider et al. 2007; Kidd et al. 2008; Huet et al. 2022). For CI users, some studies have reported a benefit from gender differences between target and masker speakers (Cullington & Zeng, 2008; Misurelli & Litovsky, 2015; for CI children, Visram et al. 2012), whereas other studies did not find such a benefit (Stickney et al. 2004; Bernstein et al. 2016). Likewise, the results of a benefit from target-masker voice differences in F0 or VTL are inconclusive. Although some studies found that target-masker differences in F0 improved CI users’ perception of speech in competing speech (Pyschny et al. 2011; Meister et al. 2020), other studies did not find such an effect for F0 differences (Stickney et al. 2004, 2007; Auinger et al. 2017; El Boghdady et al. 2019) or VTL differences (El Boghdady et al. 2019). Meister et al. (2020) suggested that these discrepancies may partly be because of the differences in masker stimuli, differing in amounts of energetic and informational masking. However, other stimulus characteristics (e.g., open-set vs. closed-set speech perception), the testing parameters (e.g., different speakers, vs. artificially manipulated target-masker voice differences), and the auditory task (e.g., repetition of the complete target sentence vs. specific target words) may also impact the outcomes (Litovsky et al. 2017).

### Developmental Effects on the Perception of Voice Characteristics

Assessing the voice and speech perception abilities of prelingually deaf CI children is complicated because of combined effects of perceptual limitations posed by the degraded CI signal and cognitive-developmental factors, such as selective auditory attention and linguistic abilities (Kronenberger et al. 2013). Even in NH school-age children, these cognitive-developmental factors likely impact lower-level voice perception, such as their ability to discriminate speakers’ voices based on differences in mean F0 (Buss et al. 2017; Nagels et al. 2020a) or VTL (Nagels et al. 2020a). Nagels et al. (2020a) found that the F0 and VTL discrimination thresholds of NH school-age (4–12 years) children and their perceptual weight attributed to F0 and VTL cues for voice gender categorization gradually develop during these years. In a follow-up study with the same NH children, Nagels et al. (2021a) reported that NH children benefited from target-masker voice differences in F0 and VTL for the perception of speech in competing speech at all tested ages, although their overall accuracy continued to differ from adults. Similar to voice gender categorization, the benefit from voice gender cue differences did not relate closely to NH children’s discrimination abilities, illustrating the different developmental trajectories in these three voice-perception abilities.

For CI children, earlier studies showed that most can distinguish male from female voices at better than chance level (Osberger et al. 1991; Staller et al. 1991), whereas NH children typically show ceiling-level performance in similar tasks (Bennett & Montero-Diaz, 1982). More recently, Cleary et al. (2005) examined NH and CI children’s ability to discriminate voices by simultaneously changing a female speaker’s mean F0 and formant frequencies (similar to “VTL cues”). Although 8 of 16 tested CI children showed a response pattern similar to NH children, others did not show reliable speaker-category boundaries based on F0 and VTL differences across the stimulus continuum. These results imply that F0 and VTL cues are partially transmitted via CIs, although some CI children may not effectively utilize these cues for discrimination. Several other studies have examined CI children’s perception of acoustic and voice-related cues, such as (affective) prosody (Hopyan-Misakyan et al. 2009; Nakata et al. 2012), musical pitch and timbre (Stordahl, 2002; Jung et al. 2012; Torppa et al. 2014; Sjoberg et al. 2017), mean pitch (Kopelovich et al. 2010; Deroche et al. 2014), pitch contours (Deroche et al. 2016, 2019), or lexical tones (Barry et al. 2002; Ciocca et al. 2002; Peng et al. 2004). These studies generally indicate that most CI children exhibit lower perception scores compared with their NH peers, but their perception scores often improve with age. However, age only partly accounts for the variability in CI children’s perceptual abilities.

### Present Study

Prelingually deaf CI children face challenges with perceiving voice characteristics because of multiple factors. Their sensitivity to differences in voice characteristics may be reduced, given the perceptual limitations of the degraded CI speech signal, as implied by postlingually deaf CI adults’ limited discrimination of voice-related cues. In addition, cognitive-developmental factors most likely play a role, as evidenced by NH children’s voice discrimination development during school-age years (Buss et al. 2017; Flaherty et al. 2019; Nagels et al. 2020a) and the observed effects of chronological age on CI children’s pitch discrimination (Kopelovich et al. 2010; Deroche et al. 2014).

In this study, we aimed to investigate the perception and use of F0 and VTL in CI children, taking into account developmental effects using data from NH children and adults from previous studies (Nagels et al. 2020a, 2021a) and data collected from postlingually deaf CI adults from previous studies using similar experiments (Fuller et al. 2014; Gaudrain & Başkent, 2018; El Boghdady et al. 2019, 2021; Nogueira et al. 2021). In three experiments, we examined CI children’s voice cue discrimination (experiment 1), voice gender categorization (experiment 2), and perception of speech in competing speech and benefit from target-masker F0 and VTL differences (experiment 3), using the same experimental tasks from previous studies in the same project on the perception of indexical cues in kids and adults (Nagels et al. 2020a, 2020b, 2021a).

## EXPERIMENT 1: VOICE CUE DISCRIMINATION

### Materials and Methods

#### Participants

**TABLE 1.** Demographic characteristics of CI children (CI children N = 14)

Subject	CIK001	CIK002	CIK003	CIK004	CIK005	CIK006	CIK007	CIK008	CIK009	CIK010	CIK011	CIK012	CIK013	CIK014
**Age (y**)	12.17	14.92	10.67	8.58	14.08	4.00	5.92	6.08	9.17	6.42	16.92	11.92	11.34	11.08
**Age at first implantation (y**)	1.25	4.25	1.00	1.00	2.00	1.00	0.83	0.92	2.34	0.92	2.00	0.92	4.17	0.75
**Hearing age (y**)	10.92	10.67	9.67	7.67	12.08	3.00	5.08	5.17	6.67	5.42	14.92	11.00	7.17	10.25
**Implant(s**)	Unknown	Unknown	Unknown	Unknown	Unknown	CI24RE (AU)	CI24RE (AU)	CI24RE (AU)	CI24RE (AU)	CI24RE (AU)	Unknown	CI512 (AS)/CI24RE (AD)	CI24RE (AD)	CI512 (AS)/CI24RE (AD)
**Processor**	Unknown	CP1000	Unknown	Unknown	CP1000	CP910/920	CP1000	CP1000	CP910/920	CP1000	CP1000	CP910/920	CP910/920	CP1000
**Strategies**	Unknown	Unknown	Unknown	Unknown	Unknown	MP3000 (AU)	MP3000 (AU)	MP3000 (AU)	MP3000 (AU)	MP3000 (AU)	Unknown	ACE (AU)	MP3000 (AD)	MP3000 (AS) /ACE (AD)
**Etiologies**	CMV	Likely CMV	Genetic	Genetic	Genetic Cx26	Genetic Cx26	Genetic Cx26	Genetic	Unknown	Genetic	Genetic PS	Unknown	Auditory neuropathy	Genetic
**Vocabulary score**	37	45	43	37	46	26	35	39	37	28	46	49	31	47

Age is given in years, rounded to two decimal places.

AS (left ear), AD (right ear), and AU (both ears) describe the characteristics of the implants for each ear. For the etiologies, PS, CMV, and genetic Cx26 indicates that the cause of deafness was a mutation of the protein Cx26 that is found on the GJB2 gene. Vocabulary corresponds to scores on the Renfrew Word Finding Vocabulary Test (maximum of 50 points).

AD, auris dextra; AS, auris sinistra; AU, auris utraque; CMV, cytomegalovirus; Cx26, Connexin 26; GJB2, Gap junction beta-2; PS, Perrault Syndrome.

Fourteen prelingually deaf CI children aged between 4 and 16 years were participated. Inclusion criteria for participation were chronological age (aged 4–16), prelingual deafness (onset before age 6), overall good health, proficiency in Dutch spoken language, and at least 1 year of CI use. For CI children, the age range was extended to 16 years of age, which was used in the studies by Nagels et al. (2020a, 2021a), as even 12-year-old NH children did not always show adult-like performance yet on some voice-perception tasks. All except one child, CIK013, were congenitally deaf. CIK013 had deafness in one ear and profound hearing loss in the other ear from the age of 3, caused by cytomegalovirus. The child used a hearing aid in the better ear until they became bilaterally deaf 1 year later and received 2 CIs. All CI children except CIK002 were bilaterally implanted. Parental questionnaires completed for 12 of 14 CI children, revealed that 9 children wore hearing aids before implantation, typically from around 3 mo of age until surgery, except for CIK002 who periodically wore a hearing aid until age 10 and CIK013 (mentioned earlier). All participants primarily communicated in oral Dutch. Two CI children also regularly communicated using Dutch Sign Language and 3 CI children also used Sign-supported Dutch. Vocabulary size was assessed using the Dutch version of the Renfrew Word Finding Vocabulary Test (Renfrew, 1995) featuring 50 line-drawn pictures (maximum of 50 points). The CI children’s demographics and hearing history can be found in Table 1. Compared with NH children’s vocabulary scores per age group (see Table 1 in Nagels et al. 2021b), 4 CI children had vocabulary scores equal to or above the NH mean, 4 CI children had scores below the NH mean but within the NH range, and 6 CI children had scores outside the NH range.

The age at implantation of CI children for their first implant ranged from 9 mo to 51 mo of age, with a median age of 12 mo. For some analyses, we added supplemental materials considering CI children’s hearing age instead of their chronological age. Hearing age is defined here as the difference between CI children’s chronological age and their age at first implantation, that is, time after CI implantation. CI children’s hearing age takes the period of auditory deprivation into account, and thus may give a fairer comparison to NH children than chronological age.

The control group consisted of 58 Dutch native-speaker NH children between 4 and 12 years of age, and 15 NH adults between 20 and 29 years of age who participated in the studies by Nagels et al. (2020a, 2021a) using the same experiments, published as part of the perception of indexical cues in kids and adults project. Adult participants, the parents and legal guardians of NH and CI children, and NH and CI children older than 12 provided written informed consent, in agreement with the regulations of the Dutch Medical Research Involving Human Subjects Act (WMO). The study protocol was approved by the Medical Ethical Review Committee of the University Medical Center Groningen (METc 2016.689).

#### Stimuli and Apparatus

For this experiment, the same stimuli and resynthesis procedure were used as in the study by Gaudrain and Başkent (2018) with postlingually deaf CI adults. Stimuli were created from 61 CV syllables extracted from Dutch CVC words of the speech corpus of Nederlandse Vereniging van Audiologie (NVA) (Bosman & Smoorenburg, 1995). Each trial involved randomly selecting and concatenating 3 CV syllables to create trisyllabic CVCVCV pseudowords, for example, *ba-ki-mo*. The recordings were produced by a female Dutch native speaker, with a mean F0 of 242 Hz and an estimated VTL of 13.5 cm based on the average height of Dutch women of 168.72 cm (Roser et al. 2020) and the regression between VTL and speakers’ height reported by Fitch and Giedd (1999). Even though mean F0 differences are commonly expressed in Hertz and VTL differences in centimeters, changes in both voice cues result in frequency differences. Hence, we expressed the applied F0 and VTL differences as ratios on a logarithmic scale in semitones (st). This approach aligns with previous studies (Fuller et al. 2014; Gaudrain & Başkent, 2018; El Boghdady et al. 2019, 2021; Nagels et al. 2020a, 2021a; Nogueira et al. 2021) and centers NH adults’ mean F0 and VTL discrimination thresholds between 1 and 2 st.

We used STRAIGHT software (Kawahara & Irino, 2005) in Matlab to implement the voice differences in F0 and VTL. The CV syllables were first analyzed to extract the F0 contour and spectral envelope, equalized for root-mean-square level, and normalized to a duration of 200 ms and a mean F0 of 242 Hz. For each trial, the F0 contour and spectral envelope of 3 randomly selected CV syllables were resynthesized with the new F0 and VTL parameters using STRAIGHT. We performed this resynthesis procedure even for the reference pseudowords to ensure no perceptual differences would result from the resynthesis procedure itself. The 3 CV syllables were subsequently concatenated with 50 ms of silence between them. The mean F0 of each syllable was changed to the intended value by multiplying the F0 contour (in hertz) by the appropriate factor, thus preserving existing F0 fluctuations. In addition, the overall F0 contour across syllables was modified by increasing or decreasing the mean F0 of each syllable by random steps of ±⅓ st (1.9%) to make the pseudowords sound less artificial. The VTL of the pseudowords was adjusted by compressing the spectral envelope linearly toward lower frequencies, whereas retaining the formant frequency ratio, equivalent to a uniform shift on a logarithmic frequency axis. After these manipulations, we recombined the modified F0 contour and spectral envelope using STRAIGHT’s pitch synchronous overlap-add (PSOLA) resynthesis method. For more details and visualizations of the effects of this voice-manipulation procedure on acoustic signals, see Gaudrain and Başkent (2018).

We used an identical CVCVCV pseudoword structure for both the target and the 2 reference pseudowords in each trial. The target pseudoword differed from the 2 reference pseudowords in either the mean F0 or VTL, with the F0 contour consistent across intervals. Depending on the measured JND, the target pseudoword either had a lower F0 or a larger VTL, making it sound more male-like than the reference pseudowords.

#### Procedure

Participants’ just-noticeable differences (JNDs) in F0 and VTL cues were measured via a 3-interval 3-alternative forced-choice (3I-3AFC) adaptive procedure. Participants first performed 2 practice sessions, each of which included 3 trials, to familiarize themselves with the task. The practice sessions were followed by 2 experiment sessions, which consisted of approximately 25 to 45 trials depending on participants’ responses. The total duration of the experiment was around 15 minutes.

The order in which the F0 or VTL JNDs were measured was randomized for each participant. Each experiment session started with a voice difference of –12 st in F0 or +12 st in VTL. Participants’ JND was determined using a 2-down 1-up adaptive staircase procedure. The voice difference would decrease with 1 step size after 2 successively correct responses and would increase with 1 step size after an incorrect response. The initial step size value was 2 st, but it was reduced by √2 after 15 consecutive trials with the same step size or when the difference became smaller than twice the step size. The experiment session concluded after 8 reversals and the geometric mean of the voice-difference values from the last 6 reversals was calculated, corresponding to the 70.7% discrimination point of the psychometric function (Levitt, 1971).

The experiment was performed on a Dell XPS 13 inch touchscreen laptop via a child-friendly game interface that was created in Matlab (Nagels et al. 2020a). In each trial, 3 sea animals appeared on the screen and sequentially produced the same CVCVCV-structured pseudoword. Two of them produced the reference pseudoword, whereas one produced the target pseudoword with a different F0 or VTL value. Participants were instructed to click on the sea animal whose voice differed from the other 2 sea animals. Visual feedback was provided to participants, with a red box appearing around the selected sea animal for incorrect responses and the sea animal going into 1 of 7 bubbles for correct responses.

The experimental setup was kept consistent for all 3 experiments. Stimuli were presented via Sennheiser HD 380 Pro headphones for NH children and adults, and via Logitech Z200 loudspeakers for CI children. The 3 experimental tasks were all performed on the same day and location during 1 test session of approximately 60 to 90 minutes. NH and CI children were visited by the experimenters and tested in a quiet room at their homes, and NH adults were tested in a quiet testing room at the University of Groningen. We instructed CI children to use their usual device settings during all experiments. The presentation level of the stimuli was calibrated to a sound level of 65 dBA for all experiments.

#### Data Analysis

We examined if CI children’s ability to discriminate differences in F0 and VTL cues by analyzing their log-transformed F0 and VTL JNDs. Because of the specifics of our adaptive procedure, applying log-transformation to JNDs expressed in semitones improves data normality, as was shown in previous studies (e.g., El Boghdady et al. 2019; Nagels et al. 2020a). In addition, we were interested in the development of these JNDs across age. Age was used as a continuous variable for data analysis, but 6 age groups were used in the figures spanning 2 to 3 years each (4–6 years, 6–8 years, 8–10 years, 10–12 years, 12–14 years, and 14–17 years, with, for instance, the 4 to 6 age group including children age 4 or older but younger than 6) for clearer visualization. For data analysis, we reported the results of CI children using their chronological age. The same figures using CI children’s hearing age are shown in Supplementary Materials http://links.lww.com/EANDH/B344. Replacing chronological age with hearing age shifted CI children’s JNDs to NH-age-appropriate values without significantly altering the outcomes.

To address potential type II errors because of our relatively small CI user sample, we evaluated where CI users fell within the distribution of JNDs in the NH group (where quantiles are cubic splines as a function of age). For this purpose, we used a quantile regression based on generalized additive models using the *qgam* package (version 1.3.4, Fasiolo et al. 2021) in R (version 4.0.3, R Core Team, 2020). The advantage of using generalized additive models over traditional linear mixed-effects models is that we can model linear and nonlinear relationships between the response and predictors. Moreover, the fitting method in *qgam* is nonparametric, and hence does not have the assumption of normality. The *qgam* method also automatically estimates the optimal dimensionality of the splines required to model the quantiles. We included data of NH children and adults and interpolated between ages 12 and 20, although we marked this age gap with hatches where NH measurements were lacking.

In the figures later, we show the estimated percentiles of the distribution of JNDs for NH listeners by age and overlay the JNDs of CI children. This comparison reveals the deviation of CI children’s F0 and VTL JNDs from the NH distribution. In addition, we included data from postlingually deaf CI adults, aged 47 to 74, from the study by Gaudrain and Başkent (2018), assigning an arbitrary age of 24.7 for the quantile regression, equivalent to the mean age of the NH adults, to attain a percentile estimate. The voice discrimination experiment in the current study differed from that of Gaudrain and Başkent (2018) in test duration. Although they used 3 repeated JND measurements per condition per participant, only 1 measurement per voice condition was obtained in this study to keep the experiment duration more appropriate for children. Moreover, although the same random F0 contour was applied to the 3 intervals in each trial in the present study, Gaudrain and Başkent (2018) randomized the contour across intervals.

### Results

**Fig. 1.**
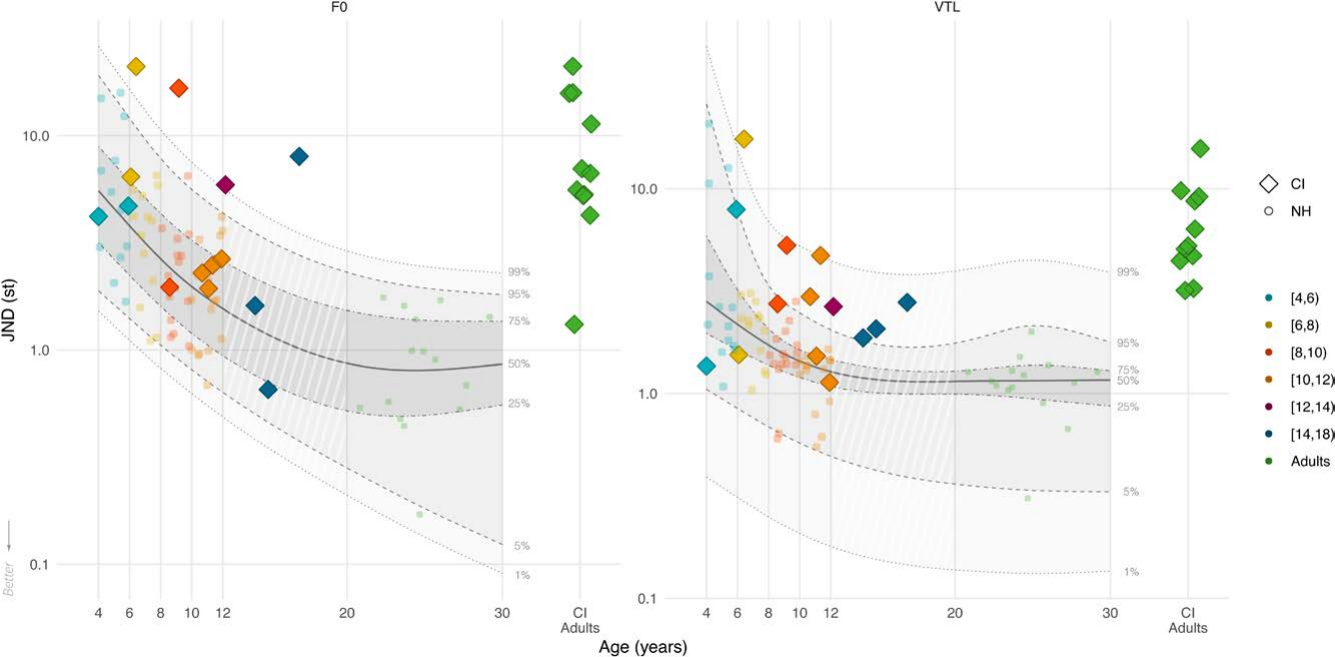
Voice JNDs (smaller indicates better discrimination) of CI children (color-coded diamonds), CI adults (green diamonds, on the right side of each panel), and NH children and adults (color-coded circles) for F0 (left panel) and VTL (right panel) as a function of participants’ chronological age (CI children N = 14; CI adults N = 11; NH children N = 58; NH adults N = 15). The colored diamonds and circles show individual data points of CI children, NH children, and NH adults located at their chronological age in years, rounded to 2 decimal places. The green diamonds show the JNDs of the postlingually deaf CI adults who were previously tested by Gaudrain and Başkent (2018). Note that the data of CI adults is not placed at the correct place on the *x* axis, and some random jitter was added to minimize visual overlap. The solid lines show the median (50th percentile) of the quantile regression for NH data. The shaded areas, and the dashed and dotted lines show the various estimated percentiles based on the regression. CI indicates cochlear implant; JNDs, just-noticeable difference; NH, normal-hearing; VTL, vocal-tract length.

Figure 1 shows the F0 and VTL JNDs of CI children (color-coded diamonds by age groups) within the JND distribution of NH children (color-coded circles) and NH adults (green circles) by chronological age (see Figure, Supplemental Digital Content 1 http://links.lww.com/EANDH/B340, which shows the same figure using hearing age). The shaded areas show, from bottom to top, the 1st, 5th, 25th, 50th, 75th, 95th, and 99th percentiles of the quantile regression using data from NH participants. In addition, data from 11 CI adults tested using a similar procedure by Gaudrain and Başkent (2018) (green diamonds) are displayed in Figure 1 for visual comparison. We can visually observe that the JNDs of most CI children were above the median JND values of the NH distribution.

For F0, 4 out of 14 CI children had JNDs above the 99th percentile based on their chronological age, determined through the quantile regression using NH data, whereas all but one CI adult had F0 JNDs surpassing the 99th percentile. Other CI children had F0 JNDs that were clustered around the median NH JND estimates, although overall above the median (50th percentile). For VTL, most CI children had JNDs below the 99th percentile, whereas those of CI adults were clustered around and above the 99th percentile. However, 10 out of 14 CI children had VTL JNDs above the 75th percentile, with only 4 CI children having JNDs below this threshold, indicating that most CI children have considerably higher VTL JNDs compared with the NH JND values. Thus, more CI children had JNDs surpassing the upper quartile (75th percentile) for VTL than F0. Comparing the JNDs of CI children to those of CI adults, we can observe that 9 out of 14 CI children had smaller F0 JNDs and 4 out of 14 CI children had smaller VTL JNDs than the 75th percentile of the NH distribution. Conversely, CI adults mostly had F0 and VTL JNDs exceeding the 99th percentiles.

### Discussion

Our results show that CI children’s F0 and VTL JNDs were overall higher than those of their NH-age-equivalent peers. However, a few CI children still showed JNDs that were approximately within the NH-age-appropriate range. CI children also generally had lower age-adjusted F0 and VTL JNDs than the CI adults tested by Gaudrain and Başkent (2018), who mostly had F0 and VTL JNDs beyond the 99th percentile. Finally, more CI children had JNDs that were within the upper quartile (75th percentile) for F0 than for VTL.

The overall higher JNDs for CI children than for NH children align with previous studies on pitch discrimination using nonvoice stimuli (Kopelovich et al. 2010; Deroche et al. 2014) and speaker discrimination based on simultaneous changes in F0 and VTL cues (Cleary et al. 2005). Similar to these studies, chronological age could not explain the observed differences in the F0 and VTL discrimination thresholds of CI children as well as it does for NH children. Most CI children had higher discrimination thresholds than their NH-age-equivalent peers, but some CI children had discrimination thresholds that were close to the NH median. This variability among the discrimination thresholds of CI children suggests that F0 and VTL cues are delivered to a certain degree by their CI, but either the level of fidelity differs across CI users or some CI children may better utilize these cues than others.

Prelingually deaf CI children demonstrated notably better sensitivity to small differences in voice cues compared with postlingually deaf CI adults tested by Gaudrain and Başkent (2018). Although postlingually deaf CI adults had mean JNDs of 9.19 st (70.7%) for F0 and 7.19 st (51.5%) for VTL, prelingually deaf CI children had mean JNDs of 5.92 st (40.8%) for F0 and 4.10 st (26.7%) for VTL. Furthermore, fewer CI children deviated from the NH distribution for F0 than for VTL, unlike CI adults. This observation aligns with the study by Zaltz et al. (2018), which linked early exposure to the spectrotemporally degraded CI signal to better VTL perception. In addition, peripheral factors related to deafness cause (primarily genetic in CI children), or device-related factors, such as differences in CI stimulation strategies (Wouters et al. 2015) and bilateral implantation (Litovsky et al. 2006; Sparreboom et al. 2021), may also play a role, as discussed further in the general discussion section.

## EXPERIMENT 2: VOICE GENDER CATEGORIZATION

### Materials and Methods

#### Participants

The same participants from the first experiment participated in the second experiment, except for the youngest CI child (4 years old) who did not fully complete the second and third experiments.

#### Stimuli and Apparatus

We used the same stimuli and resynthesis procedure for the voice gender categorization experiment as Fuller et al. (2014). The stimuli consisted of 4 CVC words, *bus* [bus], *vaak* [often], *leeg* [empty], and *pen* [pen], taken from the same NVA corpus recordings (Bosman & Smoorenburg, 1995) and produced by the same female speaker as in experiment 1.

We manipulated the F0 and VTL parameters and resynthesized all stimuli using the same STRAIGHT procedure as in experiment 1. The experiment included 9 voice conditions, combining 3 F0 parameters of 0 st, –6 st, and –12 st, corresponding to mean F0 values of 201 Hz, 142 Hz, and 100 Hz, with 3 VTL parameters of 0 st, 1.8 st, and 3.6 st, corresponding to estimated VTL sizes of 13.5 cm, 15.1 cm, and 16.6 cm. The F0 and VTL parameter values were derived from Fuller et al. (2014) and supported by earlier studies by Peterson and Barney (1952), Smith and Patterson (2005), and Smith et al. (2007). Fuller et al. (2014), using 5 different F0 parameters and 6 VTL parameters, confirmed that a combined change of –12 st in F0 and 3.6 st in VTL reliably made the speaker’s voice sound male to NH adult listeners.

#### Procedure

Participants performed the voice gender categorization experiment after completing the voice JND experiment. The experiment consisted of a visual-auditory match-to-sample task measuring participants’ perceptual weight attributed to F0 and VTL differences. Participants were familiarized with the task via a practice session of 5 trials using 5 randomly selected items. The experiment consisted of 36 trials, that is, 4 items (1 item per CVC word) per voice condition, presented in a randomized order, with a total duration of approximately 6 minutes.

A child-friendly interface was used to administer the experiment in Matlab (Nagels et al. 2020a). Participants heard an auditory stimulus resembling a female, male, or somewhat ambiguous gender voice, depending on the F0 and VTL parameters of the voice condition. Afterward, a male or female face would appear on the screen. Participants were instructed to click on the green checkmark when the gender of the voice and face matched, and on the red cross for a mismatch. Participants were informed that in some cases the gender may be ambiguous. No feedback was given.

#### Data Analysis

For the analysis of participants’ weighting of F0 and VTL cues for voice gender categorization, we calculated their *cue weights*. We computed a mixed-effects logistic regression model using the lme4 package (version 1.1.27.1, Bates et al. 2015) in R with *response* (“woman” corresponding to 1, “man” corresponding to 0) and random intercepts and slopes for δF0 and δVTL per participant. We extracted the coefficients from this model, which are predictive values on a logit scale and converted the coefficients to Berkson units per semitone (Bk/st) by scaling the factors to correspond to log-base-2 odds ratios instead of natural logs (Hilkhuysen et al. 2012). An increase of 1 Bk/st corresponds to doubling the ratio of “woman” to “man” categorizations for each semitone of voice difference. We then applied the same quantile regression analysis on these cue weights as was used for the JNDs. In addition, the cue weights of 19 CI adults from Fuller et al. (2014) who used the same F0 and VTL voice resynthesis procedure are plotted (green diamonds). Note that the experimental task that was used by Fuller et al. (2014) differed from our task in a few minor details. CI adults had to specify if the voice was from a woman or a man, whereas CI children had to specify if the gender of the voice and displayed face matched. Although Fuller et al. (2014) used a finer grid of F0 and VTL values, this is expected to minimally affect cue weights obtained through logistic regression. These differences did not alter F0 and VTL ratios for NH adults, as seen in Fuller et al (2014) and Nagels et al. (2020a).

In addition, to investigate the relationship between discrimination abilities (JNDs) and cue weights, we calculated the Pearson correlation coefficients of the probit-transformed quantile representations of the data for each voice cue. To clarify, each data point was expressed as a quantile of the NH distribution and probit-transformed to obtain the underlying probability unit (in number of standard deviations). This approach neutralizes any common age-related effects in the correlated measures, akin to “partialling out” age in a linear model but without assuming a linear age trajectory.

### Results

**Fig. 2.**
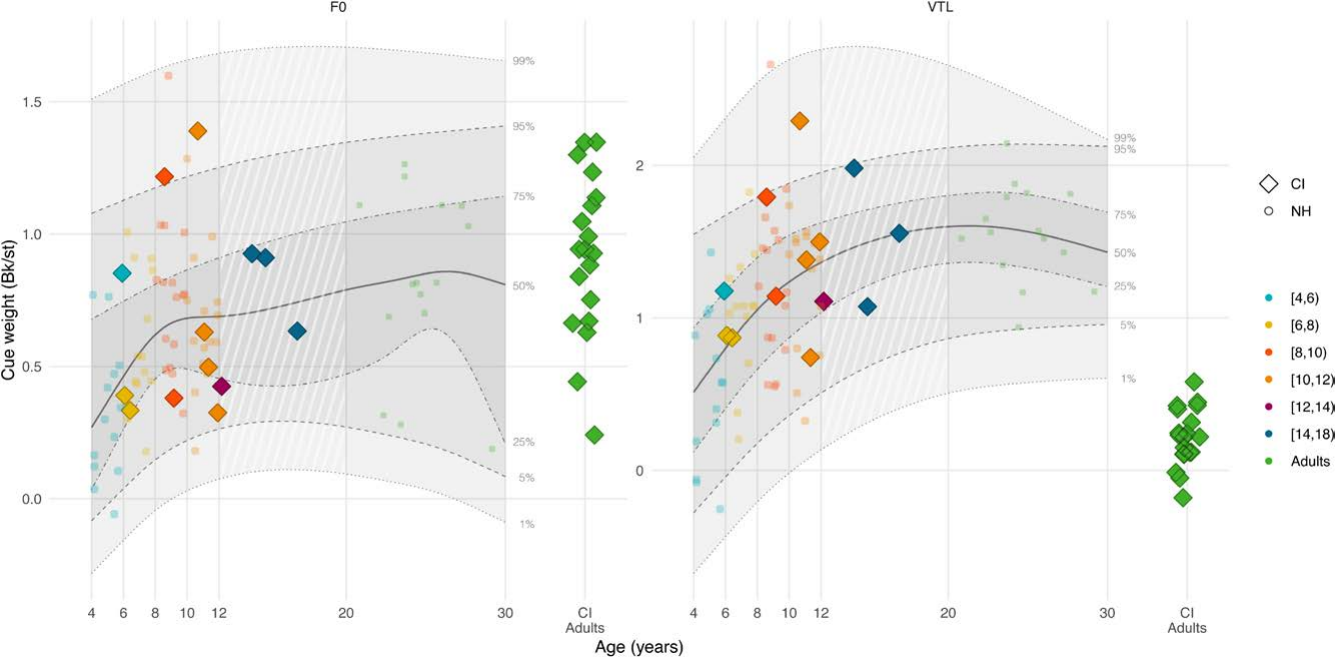
Voice gender categorization cue weights in Bk/st of CI users (color-coded diamonds) and NH listeners (color-coded circles) for F0 (left panel) and VTL (right panel) as a function of their chronological age (CI children N = 13; CI adults N = 19; NH children N = 58; NH adults N = 15). The CI adult data (green diamonds on the right-hand side of each panel) are reproduced from Fuller et al. (2014). The solid lines show the median (50th percentile) of the quantile regression for NH data. The shaded areas, and the dashed and dotted lines show the various estimated percentiles based on the regression. CI indicates cochlear implant; F0, fundamental frequency; JNDs, just-noticeable difference; NH, normal-hearing; VTL, vocal-tract length.

Figure 2 illustrates the F0 and VTL cue weights of CI children in Bk/st units (color-coded diamonds by age groups) and how they fit within the distribution of cue weights of NH children and adults (color-coded circles) based on their chronological age (see Figure, Supplemental Digital Content 2 http://links.lww.com/EANDH/B341, which shows the same figure using hearing age). The shaded areas show, from bottom to top, the 1st, 5th, 25th, 50th, 75th, 95th, and 99th percentiles of the quantile regression derived from NH participant data. Notably, only 2 CI children had F0 cue weights exceeding the 95th percentile and only 1 CI child had a VTL cue weight exceeding the 95th percentile. These CI children attributed more weight to F0 and VTL differences than their NH-age-equivalent peers. The F0 and VTL cue weights of the other CI children all fell within the 5th and 95th percentiles. The cue weights of CI adults all fell within the 25th and 95th percentiles for F0 but not for VTL. For VTL, almost all CI adults had VTL cue weights below the 1st percentile, showing that they attributed far less weight to VTL differences for voice gender categorization than NH adults.

**Fig. 3.**
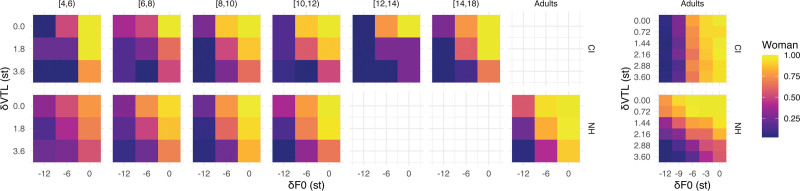
The voice gender categorization subjective judgments of CI children (upper panels), and NH children and adults (lower panels) averaged per participant age group (CI children N = 13; NH children N = 58; NH adults N = 15), and those of the postlingually deaf CI adults (upper right panel) and NH adults (lower right panel) who were previously tested by Fuller et al. (2014) as a function of differences in F0 (*x* axis) and VTL (*y* axis) (CI adults N = 19; NH adults N = 19). Dark/blue corresponds to 100% “man” categorizations and light/yellow corresponds to 100% “woman” categorizations. CI indicates cochlear implant; F0, fundamental frequency; NH, normal-hearing; VTL, vocal-tract length.

In addition, Figure 3 shows voice gender categorization as a function of F0 and VTL manipulations displayed as a matrix, showing the ratio of “man” and “woman” categorizations per voice combination. CI children, like NH children, assign weight to both F0 and VTL cues by showing categorization patterns resembling those of NH children and adults. These patterns differ from those of CI adults tested by Fuller et al. (2014), who exhibited a relatively vertical pattern, indicating a greater reliance on F0 differences (*x* axis) than VTL differences (*y* axis) for voice gender categorization.

Finally, we calculated the Pearson correlation coefficients between the probit-transformed quantiles of CI children’s cue weights and their JNDs for each voice cue. For F0, we found a significant negative correlation between CI children’s JND quantiles (from experiment 1) and cue weight quantiles (*r*_11_ *=* –0.56, *p <* 0.05), indicating that CI children with higher-than-normal F0 JNDs, relative to NH children, had lower-than-normal F0 cue weights. However, there was no significant correlation for VTL (*r*_11_ *=* –0.07, *p =* 0.81).

### Discussion

The results from the second experiment revealed that CI children’s perceptual weight attributed to F0 and VTL cues for voice gender categorization did not differ from NH children. Unlike postlingually deaf CI adults, who mainly rely on F0 differences and use VTL cues to a lesser extent (Fuller et al. 2014; Meister et al. 2016; Skuk et al. 2020), CI children relied on both F0 and VTL cues for voice gender categorization. For both NH and CI children, the cue weights increased to adult-like levels during the school-age years. Finally, a significant negative correlation was observed between CI children’s F0 JNDs and F0 cue weights, indicating that CI children with higher-than-normal F0 discrimination thresholds attributed less weight to F0 differences than NH children for voice gender categorization.

Most CI children in our study consistently categorized voices at the outer ends of the stimulus continuum as “woman” or “man,” which appears to differ from earlier studies (Osberger et al. 1991; Staller et al. 1991; Cleary et al. 2005). This discrepancy may be because of the lower median age at implantation of CI children, which was 12 mo in the present study and 36 mo in the study by Cleary et al. (2005), as age of implantation can affect perceptual outcomes and VTL perception (Zaltz et al. 2018). The different task demands, speaker discrimination versus voice gender categorization, may have contributed to the different outcomes, as similar inconsistencies have been observed in NH adult listeners (Johnson et al. 2020; Lavan et al. 2020). Moreover, we used larger step sizes than those applied by Cleary et al. (2005) of 0.5 st, which may have made the voice gender differences more noticeable to CI children. Finally, Cleary et al. (2005) manipulated F0 and VTL concurrently instead of independently and had a maximum difference of 6 st, which was less than the 12 st F0 difference and more than the 3.6 st VTL difference in the present study.

The results from experiment 2 are consistent with experiment 1 in showing that CI children are better able to perceive and use the reduced F0 and VTL cues than CI adults. In earlier studies on phoneme categorization, CI children showed cue weighting strategies resembling those of NH children (Giezen et al. 2010), whereas those of CI adults often differed from NH adults (Moberly et al. 2014; Winn & Moore, 2018). Somewhat in line with the idea of CI children having more robust voice representations than CI adults, Meister et al. (2016) found that postlingually deaf CI adults show more reliance on VTL cues for voice gender categorization when sentence stimuli instead of word stimuli are used, although still to a lesser degree than NH adults. Sentence stimuli provide diverse phonetic and suprasegmental cues, which may facilitate F0 and VTL processing in CI users. Together, these findings suggest that whereas prelingually deaf CI children can already efficiently extract this information from short word stimuli, postlingually deaf CI adults may require more processing time or more acoustic information, or possibly training, to make use of VTL cues. However, peripheral limitations may still affect the perceptual weight that CI children attributed to F0 differences as is shown by the significant correlation with their F0 discrimination thresholds.

## EXPERIMENT 3: SPEECH PERCEPTION IN COMPETING SPEECH

### Materials and Methods

#### Participants

The same participants from the previous experiments also performed the third experiment except for the same 4-year-old CI child who also did not complete experiment 2 and three 4-year-old NH children who had partial participation because of attentional and motivational reasons. The speech-in-competing-speech perception task was conducted last and was relatively long with a duration of 15 to 20 minutes. For data analysis, we excluded the partial data from the CI child and 3 NH children who did not fully complete the task.

#### Stimuli and Apparatus

We used a coordinate response measure (CRM, used earlier by, for instance, Moore, 1981; Bolia et al. 2000; Brungart, 2001; Hazan et al. 2009; Saleh et al. 2013; Welch et al. 2015), which was also used in the study by Nagels et al. (2021a). The sentence stimuli were adapted from the English stimuli of Hazan et al. (2009) and Welch et al. (2015), and translated to Dutch. Sentences consisted of a carrier phrase with a color, number, and the call sign (dog or cat), for examples, *Laat de hond/kat zien waar de rode* (color) *twee* (number) *is.* [Show the dog/cat where the red (color) two (number) is.] We used 6 disyllabic color words (*rode*, *zwarte*, *groene*, *blauwe*, *witte*, and *gele*) [red, black, green, blue, white, and yellow] and 8 monosyllabic number words (1–10; but excluding *zeven* [seven] and *negen* [nine], which are disyllabic words in Dutch), creating 48 sentences per call sign. All stimuli were recorded in an anechoic room by a different speaker than the NVA corpus speaker of experiments 1 and 2. The CRM speaker was a female Dutch native speaker with no discernable regional accent. The mean F0 value of the stimuli was 242 Hz, and the estimated VTL was 13.4 cm based on the speaker’s height of 166 cm and the regression between VTL and speakers’ height as reported by Fitch and Giedd (1999). The duration of the sentences was 2.27 seconds on average and ranged from 2.14 to 2.49 seconds.

To create single-talker nonsense maskers, the same sentence-chunking and voice-manipulation procedures were used as in the studies by El Boghdady et al. (2019, 2021) and Nogueira et al. (2021). Sentence chunks ranging from 150 to 300 ms were randomly selected and cut from the sentences with a cat call sign, 50 ms raised cosine ramps were applied, and concatenated. The masker speech started 750 ms before the onset and ended 250 ms after the offset of the target speech. To introduce voice differences in the F0 and VTL between the target and masker, the same STRAIGHT resynthesis procedure was applied, resulting in 4 masker voice conditions: (1) no differences, (2) –12 st F0, (3) +3.8 st VTL, or (4) –12 st F0 and +3.8 st VTL (equal to a male-sounding voice). These differences resulted in mean F0 values of 242 Hz and 121 Hz and estimated VTL values of 13.6 cm and 16.7 cm.

#### Procedure

The CRM-experiment was performed after the completion of the first 2 experiments. The target-to-masker ratios (TMRs) were selected based on Nagels et al. (2021a) and refined after the initial assessment of the CI children. Participants first performed an 8-trial practice session, with 3 trials without the competing speech masker, and 5 trials with a +6 dB TMR and target-masker voice differences in F0 and VTL. Following, the participants performed the main experiment, consisting of 84 trials with 7 items per voice condition and per TMR (0 dB, +6 dB, and +12 dB) for the first 3 tested CI children, and 112 trials for the other CI children (7 items × 4 voice conditions × 4 TMRs). Initially, we replaced the –6 dB TMR that was used by Nagels et al. (2021a) with a +12 dB TMR to include a relatively easy condition, but the first 3 CI children showed unexpectedly high accuracy scores, leading us to add a –6 dB TMR condition. The level of the target and masker mix was calibrated to a fixed level of 65 dBA. All items were presented in a randomized order within a single block, with 2 optional breaks, lasting approximately 15 to 20 minutes.

Participants were instructed to pay attention to the color and number that were mentioned in the target sentence, then select the correct color-number combination button on the touchscreen as fast as possible. Participants received 1 point for both correct color and number, and 0 points for any incorrect response, with a 2.08% chance of a random correct response. No feedback was provided.

#### Data Analysis

The experiment aimed to evaluate CI children’s overall perception of speech in competing speech, to measure their benefit from target-masker voice differences in F0 and VTL, and to determine if these abilities resembled those of NH children and the postlingually deaf CI adults tested in previous studies (El Boghdady et al. 2019, 2021; Nogueira et al. 2021). We calculated the average scores across all voice conditions per TMR. The same quantile regression method was then applied to logit-transformed data and then plotted back on a percentage scale. Saturated scores of 0% and 100%, that would lead to infinite logit values, were replaced with 0.5% and 99.5%. Note that these substituted values were closer to the saturated values than is typical for these analyses to minimize the bias introduced by this correction into the regression analysis.

To quantify the voice-difference benefit, we calculated the average Berkson difference in scores for ΔF0 changes, across the 2 ΔVTL values, and vice versa for ΔVTL changes. This Berkson difference was divided by the number of semitones of the considered change, to attain a benefit in Berkson per semitone (Bk/st). For this analysis, the typical correction of a half-step (i.e., in this case 0.5 points/ maximum of 7 points per voice and TMR condition = 7.1%) was applied to 0% and 100% cases to avoid infinite Berkson values. The same quantile regression technique was then applied to these benefit values.

In addition, we calculated the Pearson correlation coefficients between CI children’s F0 and VTL JNDs, expressed as probit-transformed quantiles of the NH distribution (experiment 1), their benefit from target-masker differences in F0 and VTL, and their overall accuracy scores, both expressed as quantiles of the NH distribution. This approach is equivalent to “partialling out” age effects in a linear regression model, but avoids assuming a linear age trajectory. Instead of evaluating the correlation between the JNDs and overall accuracy scores of CI children across TMRs, we selected a single TMR condition with no saturation in which there was a large voice benefit and data were collected from all included participants (therefore excluding data from the –6 dB TMR condition).

### Results

**Fig. 4.**
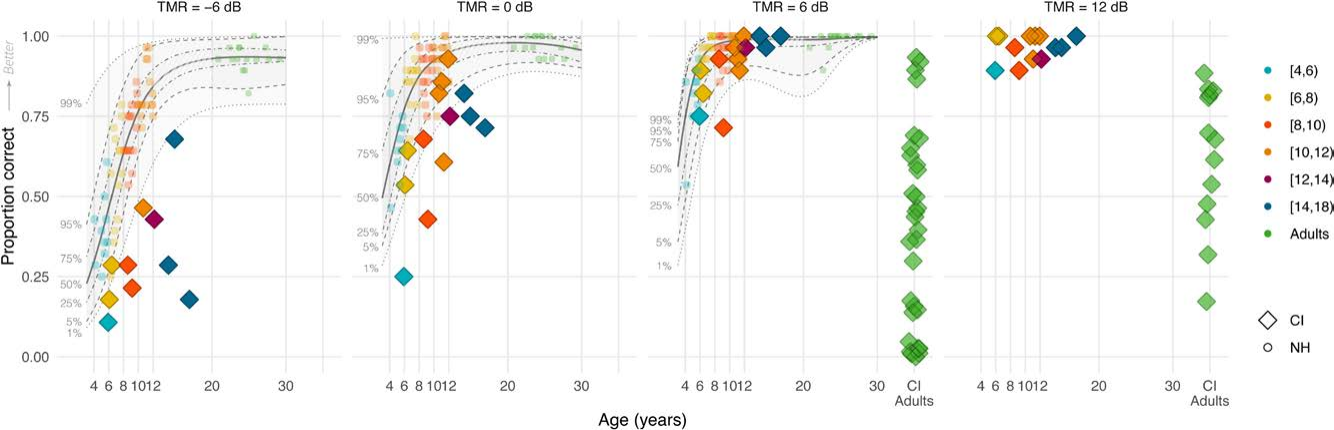
Overall accuracy scores for the CRM-experiment of CI users (color-coded diamonds) and NH listeners (color-coded circles) across all voice conditions, as a function of TMR (from left to right panel) and chronological age (CI children N = 13, NH children N = 55, NH adults N = 15, CI adults N = 43). The panels from left to right show the accuracy scores as proportion correct (0 to 1) for the –6 dB, 0 dB, and +6 dB TMR conditions for CI children, NH children, and NH adults, and the +12 dB TMR condition for only CI children. The scores from 43 CI adults who were tested in previous studies at +8 dB (El Boghdady et al. 2019; Nogueira et al. 2021) and at +10 dB TMR (El Boghdady et al. 2021) are displayed in the panel that corresponds to the closest TMR. Note that the experiment design differed from the present study (see Methods section for details). CI indicates cochlear implant; CRM, coordinate response measure; F0, fundamental frequency; NH, normal-hearing; TMRs, target-to-masker ratios; VTL, vocal-tract length.

Figure 4 shows the speech perception accuracy scores of CI children collapsed across the 4 voice conditions (color-coded diamonds by age groups) and how these fit within the distribution of NH children’s and adults’ accuracy scores (color-coded circles) in percentage points as a function of TMR and chronological age (see Figure, Supplemental Digital Content 3 http://links.lww.com/EANDH/B342, which shows the same figure using hearing age). The shaded areas denote percentiles from the quantile regression based on NH participants’ data. For visual comparison, we also included data of 18 CI adults from El Boghdady et al. (2019) using a +8 dB TMR, 13 CI adults from El Boghdady et al. (2021) using a +10 dB TMR, and 12 CI adults from Nogueira et al. (2021) using a +8 dB TMR (green diamonds, +8 dB results are plotted in the +6 dB-panel and +10 dB results in the +12 dB-panel). These studies used the same F0 and VTL voice-manipulation and single-talker nonsense maskers, with target-masker voice differences of 12 st in F0 and 3.8 st in VTL. In addition, fixed TMRs were used and participants were tasked to recollect key words from the target speech stream, as in the present study. However, some differences existed, for instance, these earlier studies used open-set sentence materials instead of closed-set CRM, and the 2 most recent of these studies were in German with German-speaking participants instead of Dutch. Also, El Boghdady et al. (2019) contrasted an adult female voice with a child voice instead of an adult male voice. These differences may affect the overall performance of CI children and CI adults across the different studies, although not necessarily their benefit from voice differences.

Visual observation shows that the accuracy scores of CI children were overall lower than those of their NH-age-equivalent peers, but primarily in the –6 and 0 dB TMR conditions and not so much in the +6 dB TMR condition. CI children older than 10 years of age approached ceiling-level performance in the +6 dB TMR condition, and all CI children achieved near ceiling performance in the +12 dB TMR condition. CI children also outperformed postlingually deaf CI adults who took part in the 3 previous studies (El Boghdady et al. 2019, 2021; Nogueira et al. 2021), although differences in experiment design and stimuli should be considered for this comparison.

**Fig. 5.**
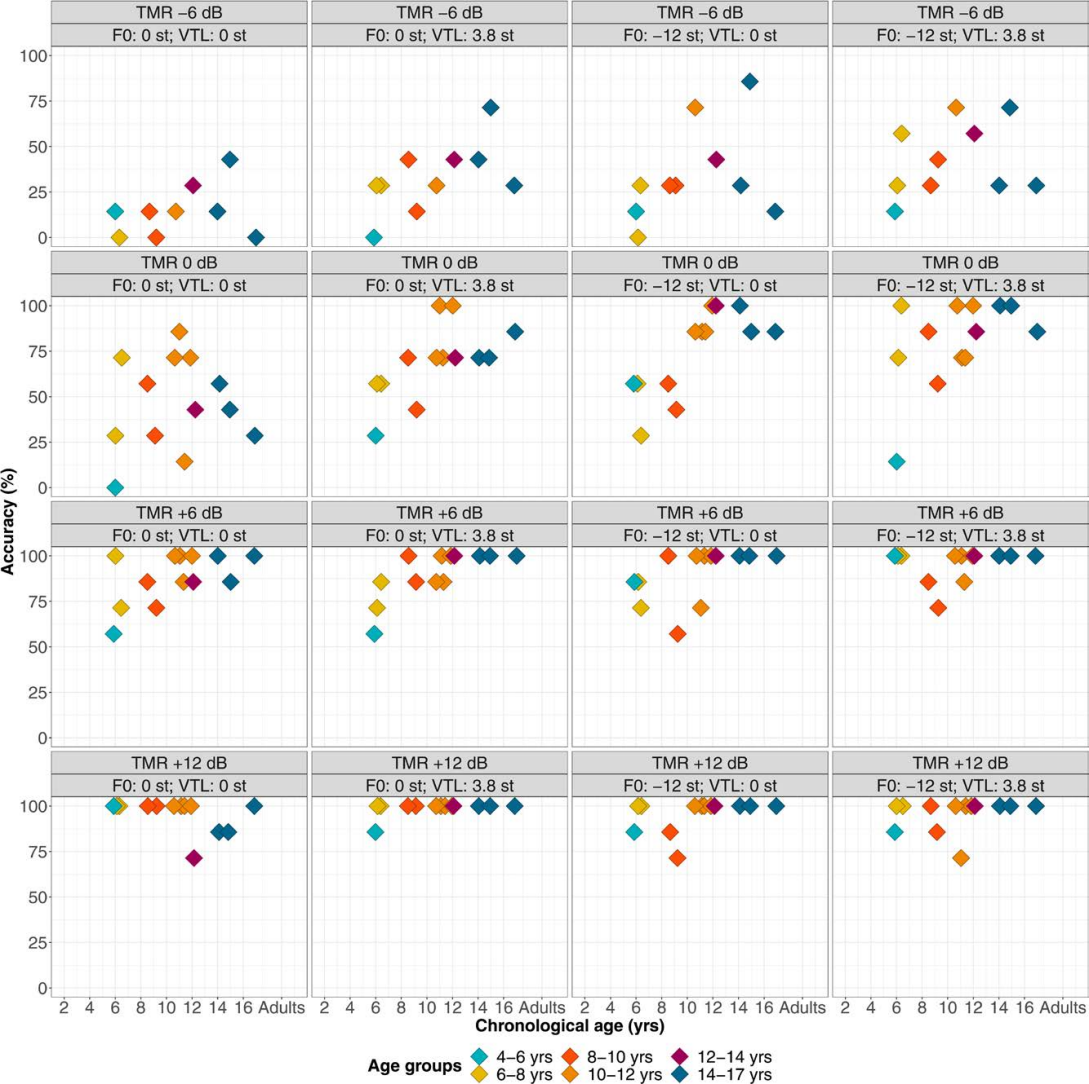
The accuracy scores of CI children for the CRM-experiment in percentage points as a function of chronological age, TMR, and voice condition (CI children N = 13). The panels from top to bottom show the accuracy scores of CI children for the –6 dB TMR (first-row panel), 0 dB TMR (second-row panel), +6 dB TMR (third-row panel), and the +12 dB TMR (fourth-row panel) conditions. Each panel consists of 4 plots that show the accuracy scores for the conditions with no target-masker differences in F0 and VTL (first-column plots), a target-masker difference of +3.8 st in VTL (second-column plots), a target-masker difference of –12 st in VTL (third-column plots), and target-masker differences of +3.8 in VTL and –12 st in F0 (fourth-column plots). CI indicates cochlear implant; CRM, coordinate response measure; F0, fundamental frequency; NH, normal-hearing; TMRs, target-to-masker ratios; VTL, vocal-tract length.

**Fig. 6.**
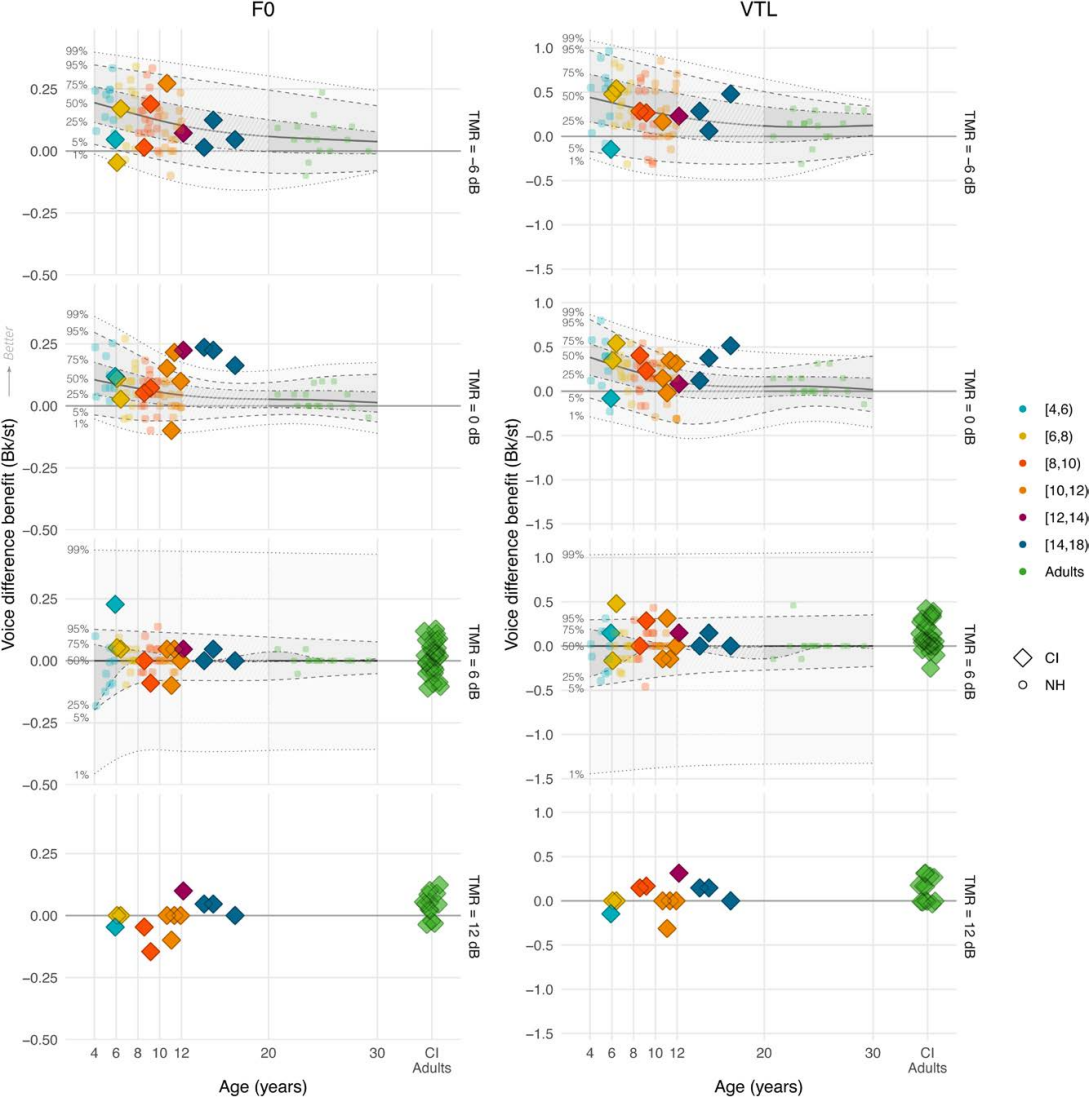
The benefit that CI users (color-coded diamonds) and NH listeners (color-coded circles) derived from target-masker voice differences in F0 (left column) and VTL (right column) in Bk/st as a function of chronological age and TMR (CI children N = 13, NH children N = 55, NH adults N = 15, CI adults N = 43). For the +12 dB TMR condition, no NH data is available and only the voice-difference benefit of CI children is shown. In the lower 2 rows, data from 43 CI adults who participated in previous studies (El Boghdady et al. 2019, 2021; Nogueira et al. 2021) are plotted. The shaded areas, and the dashed and dotted lines show the various estimated percentiles based on the regression. CI indicates cochlear implant; F0, fundamental frequency; NH, normal-hearing; TMRs, target-to-masker ratios; VTL, vocal-tract length.

Figure 5 displays CI children’s accuracy scores across TMR and voice conditions, and Figure 6 visualizes the benefit from target-masker voice differences in F0 and VTL in Bk/st (see Figure, Supplemental Digital Content 4 http://links.lww.com/EANDH/B343, which shows the same figure using hearing age). In Figure 6, a benefit of target-masker differences in F0 and VTL is characterized by values larger than 0 Bk/st. Figure 5 shows that CI children’s accuracy scores overall improved with higher TMRs and greater target-masker voice differences in F0 and VTL. CI children older than 10 years of age showed near ceiling performance in the +6 dB TMR condition with a mean accuracy score of 96.4% correct, and all CI children showed near ceiling performance in the +12 dB TMR condition with also a mean accuracy score of 96.4% correct. However, CI children mainly benefited from voice differences in the –6 and 0 dB TMR conditions, because there was less room for improvement in the other TMR conditions. Figure 6 confirms this observation, with benefits centered around 0 Bk/st for children older than 10 years of age in the +6 dB TMR condition and for nearly all in the +12 dB TMR condition. In the +12 dB TMR condition, all CI children demonstrated near ceiling performance, leaving little room for improvement in accuracy, whereas CI adults had a mean accuracy score below 50% correct (Fig. 4).

To investigate if the F0 and VTL discrimination thresholds of CI children could have limited their benefit from target-masker differences in F0 and VTL or affected their overall ability to perceive speech in competing speech, we evaluated the correlations between these measures expressed as quantiles of the NH distribution. Based on the criteria described in the data analysis section, we only selected the TMR of 0 dB for this analysis. Our results show that there was no significant correlation between CI children’s F0 JND quantiles and their corresponding quantiles for CRM benefit from F0 differences (*r*_11_ = –0.01, *P* = 0.96), and no significant correlation between their VTL JND quantiles and their corresponding quantiles for CRM benefit from VTL differences (*r*_11_ = 0.20, *P* = 0.51). There was also no significant correlation between the overall scores in the CRM task at 0 dB TMR expressed as quantiles and the F0 JND quantiles (*r*_11_ = –0.19, *P* = 0.52), or with the VTL JND quantiles (*r*_11_ = –0.52, *P* = 0.065).

### Discussion

The results of experiment 3 show that CI children’s accuracy scores improved with both higher TMRs and also greater target-masker voice differences in F0 and VTL. CI children older than 10 performed near ceiling in the +6 dB TMR condition, and all CI children performed near ceiling in the +12 dB TMR condition. Despite lower accuracy scores compared with their NH-age-equivalent peers, CI children outperformed postlingually deaf, CI adults reported in previous studies (El Boghdady et al. 2019, 2021; Nogueira et al. 2021), noting differences in experiment design and stimuli. In addition, CI children benefited from target-masker voice differences in F0 and VTL similarly to NH children, contrasting with CI adults whose benefit centered more around 0 Bk/st. Finally, we found no significant correlation between the F0 or VTL JNDs of CI children and their benefit from target-masker voice differences or overall scores.

Our findings complement those of Misurelli and Litovsky (2015), demonstrating that CI children not only benefit from differences in speakers’ genders between target and masker but also from F0 and VTL differences of the same speaker, whereas all other speaker-specific voice cues remain unchanged. This finding implies that target-masker differences in F0 and VTL cues are sufficiently represented in the degraded CI signal to allow CI children to better perceive speech in competing speech. Although CI children had higher F0 and VTL discrimination thresholds than their NH-age-equivalent peers (experiment 1), their benefit from target-masker differences in F0 and VTL and their voice discrimination thresholds were not closely related. For NH children, a similar discrepancy has also been observed (Sussman et al. 2007; Sussman & Steinschneider, 2009; Flaherty et al. 2019; Nagels et al. 2021a). The lack of a significant correlation could be caused by the relatively large target-masker differences in F0 (–12 st) and VTL (+3.8 st), which were used in this experiment. Experiments 1 and 2 showed that these differences are above the discrimination thresholds of most CI children, and CI children can also use these differences effectively for voice gender categorization. The results might differ if more subtle voice differences nearer to the discrimination thresholds were used.

Because CI children’s benefit from target-masker voice differences in F0 and VTL did not differ from NH children, other factors are likely to contribute to their overall performance in this experiment. Auditory exposure and linguistic development, such as vocabulary size, can affect children’s ability to restore masked speech segments (Corbin et al. 2016; Klein et al. 2017; McCreery et al. 2017, 2019). Given that many of the tested CI children had vocabulary scores below the NH mean and range, this factor may have diminished their speech restoration abilities. Other cognitive mechanisms, such as selective auditory attention and the inhibition of masker interference, that are involved in auditory stream segregation may also play a role (Sussman et al. 2007; Buss et al. 2017). However, based on the improving performance that we observed across the age groups tested, the perception of speech in competing speech appears to improve in CI children because they become older.

Consistent with the first and second experiments, the results of the third experiment also reveal differences in the voice-perception abilities of CI children and postlingually deaf CI adults. Although few CI adults showed a benefit from target-masker differences in F0 and VTL in previous studies, all CI children showed a benefit in the present study. These disparities may be partially attributed to differences in experiment design and stimuli across studies (Litovsky et al. 2017). For instance, Nagels et al. (2021a) found that NH school-age children benefited from target-masker differences in F0 and VTL at all tested ages, whereas Flaherty et al. (2019, 2021) reported no such benefit. In particular, using simple closed-set sentence materials with a carrier phrase may have made it easier for children to use the voice differences to segregate target and masker speech (Freyman et al. 2004; Bonino et al. 2013). Because El Boghdady et al. (2019, 2021) and Nogueira et al. (2021) used open-set sentence stimuli without a carrier phrase, these experiments may have been more difficult, although the TMR values were more favorable in these studies which may partially compensate for this difference when comparing overall performance. Combined with the results from the previous 2 experiments, our results imply that CI children may also outperform CI adults in this aspect of voice perception.

## GENERAL SUMMARY AND DISCUSSION

In the present study, we conducted 3 experiments to investigate voice discrimination, the perceptual weighting of voice cues for voice gender categorization, and the perception of speech in competing speech with varying voice differences in prelingually deaf implanted CI children. We also compared their performance to previously collected data from NH children (Nagels et al. 2020a, 2021a) and postlingually deaf CI adults (Fuller et al. 2014; Gaudrain & Başkent, 2018; El Boghdady et al. 2019, 2021; Nogueira et al. 2021). Our first experiment revealed that CI children generally had higher voice cue discrimination thresholds than their NH-age-equivalent peers, but some performed within NH ranges and most outperformed CI adults (Gaudrain & Başkent, 2018). Despite higher discrimination thresholds, the second experiment indicated that CI children weighted both F0 and VTL cues for voice gender categorization, unlike postlingually deaf CI adults, who primarily relied on F0 differences (Fuller et al. 2014; Meister et al. 2016; Skuk et al. 2020). However, CI children with high F0 discrimination thresholds showed lower F0 cue weights, indicating that lower sensitivity to F0 differences could relate to lower perceptual weighting of F0 cues for voice gender categorization. In experiment 3, CI children showed lower overall scores for perceiving speech in competing speech than their NH-age-equivalent peers, but outperformed CI adults who participated in studies by El Boghdady et al. (2019, 2021) and Nogueira et al. (2021). CI children’s accuracy scores also improved with higher TMRs and greater target-masker voice differences in F0 and VTL, similar to NH children, and unlike CI adults, of which many did not show such a benefit from F0 and VTL differences.

Although we have mostly focused on age effects on CI children’s voice-perception abilities as a group, there are several factors besides chronological age which can affect their performance. Our relatively homogeneous participant group all had similar devices and stimulation strategies (ACE or MP3000) from Cochlear Corporation, and all but one were bilaterally implanted, ruling out significant device-related differences. Underlying cause (Pyman et al. 2000) or duration of auditory deprivation (Gordon et al. 2011; Blamey et al. 2013; DiNino et al. 2020) could also play a role, although all except one CI child were congenitally deaf, mostly because of genetic factors. The list of potential external factors that could play a role is rather long, including residual hearing (Gordon et al. 2001), daily CI use (Busch et al. 2020; Gagnon et al. 2020), socioeconomic status (Niparko et al. 2010), parental involvement in the rehabilitation process (Spencer, 2004; Niparko et al. 2010; Boons et al. 2012), as well as cognitive abilities like selective auditory attention, inhibition, and auditory working memory (Kronenberger et al. 2013). Given our lengthy procedures, we did not have the opportunity to collect such information, and our participant group is too small to identify the potential influence of such predictive factors. Although age at implantation is a crucial factor for CI children’s speech and language outcomes (Kirk et al. 2002; Gaurav et al. 2020), we mainly focused on CI children’s chronological age, although we also considered hearing age in our analyses (figures available in Supplementary Materials http://links.lww.com/EANDH/B344). However, given the variability in CI children’s voice-perception abilities, a more comprehensive way of looking at age effects would be to collect longitudinal data. No such data from the literature are available yet for voice perception and speech in competing speech perception.

The results from this study show significant differences between the voice-perception abilities of prelingually deaf CI children and postlingually deaf CI adults (Fuller et al. 2014; Gaudrain & Başkent, 2018; El Boghdady et al. 2019, 2021; Nogueira et al. 2021). Based on the high VTL JNDs of CI adults (Gaudrain & Başkent, 2018), the explanation that VTL cues are not transmitted via the CI signal seemed more plausible at the time than the alternative that the distorted VTL cues prevented their utilization (Fuller et al. 2014). However, the present study supports the latter explanation that VTL cues are available in the degraded CI signal to a certain degree. Early exposure to the CI signal and brain plasticity (Manrique et al. 1999; Kral & Sharma, 2012) may have caused CI children to learn to use reduced F0 and VTL cues effectively. For CI adults, adapting existing auditory representations of speakers’ voice gender that are based on normal acoustic hearing may, in fact, impede relearning the spectrotemporally degraded voice cues and categories in the CI signal (Iverson et al. 2006; McGettigan et al. 2014; Moberly et al. 2014; Biçer et al. 2023).

Although we mainly attributed the differences between CI children and CI adults to neural plasticity and early exposure to the CI signal, additional factors need to be considered. Most of the CI adults whose data were reported from earlier studies (Fuller et al. 2014; Gaudrain & Başkent, 2018; El Boghdady et al. 2019, 2021; Nogueira et al. 2021) were older, around 60 years on average, and advanced age can impact the perception of speech in competing speech (e.g., Helfer & Freyman, 2009) and voice discrimination abilities (Zaltz & Kishon-Rabin, 2022). Another difference is that all except one CI child were bilaterally implanted, whereas most CI adults were unilaterally implanted. Even though our tasks did not rely on spatial advantages, bilateral implants provide greater access to sounds in the environment (Litovsky et al. 2006; Sparreboom et al. 2021), which could contribute to incidental learning in CI children improving speech and language development. It is also possible that CI children learn to integrate complementary information effectively from each CI device. Supporting the advantages of bilateral implantation, Boons et al. (2012) found higher scores of spoken language learning in bilaterally implanted CI children and Litovsky et al. (2006) reported higher scores for speech in noise intelligibility when bilaterally implanted CI children were tested using both CIs compared with using only 1 CI. Hence, beyond neural plasticity and learning effects, bilateral implantation might have contributed to the observed perceptual advantages in CI children by providing more exposure to voice and speech sounds in daily life.

To conclude, despite having higher F0 and VTL discrimination thresholds than their NH-age-equivalent peers, CI children showed similar cue weighting for voice gender categorization and benefited similarly from target-masker voice differences for the perception of speech in competing speech. These results highlight the role of cognitive-developmental factors on voice perception in CI children, akin to NH children, and how much the perceptual limitations imposed by the degraded CI signal can be compensated for. In addition, CI children appear to make better use of reduced F0 and VTL cues in the degraded CI signal than postlingually deaf CI adults. These findings also support the idea of greater perceptual learning in CI children than in CI adults, possibly related to early exposure to spectrotemporally degraded speech and extensive neuroplastic changes during childhood. Furthermore, these findings imply that CI signals likely transmit voice cues better than studies with CI adults have shown. Targeted rehabilitation or training approaches for CI adults could possibly also allow them to learn to make more effective use of these cues.

## ACKNOWLEDGMENTS

This work was funded by the Center for Language Cognition Groningen (CLCG), a VICI Grant from the Netherlands Organization for Scientific Research (NWO) and the Netherlands Organization for Health Research and Development (ZonMw) (Grant No. 918-17-603), the Medical Research Council (Senior Fellowship Grant S002537/1), a National Institute of Health Research Programme Grant (NIHR201608), and the Heinsius Houbolt Foundation. This work was conducted in the framework of the LabEx CeLyA (“Centre Lyonnais d’Acoustique,” ANR-10-LABX-0060/ANR-11-IDEX-0007) operated by the French National Research Agency, and is also part of the research program of the UMCG Otorhinolaryngology Department: Healthy Aging and Communication. There are no conflicts of interest, financial or otherwise.

## Supplementary Material



**Figure:**
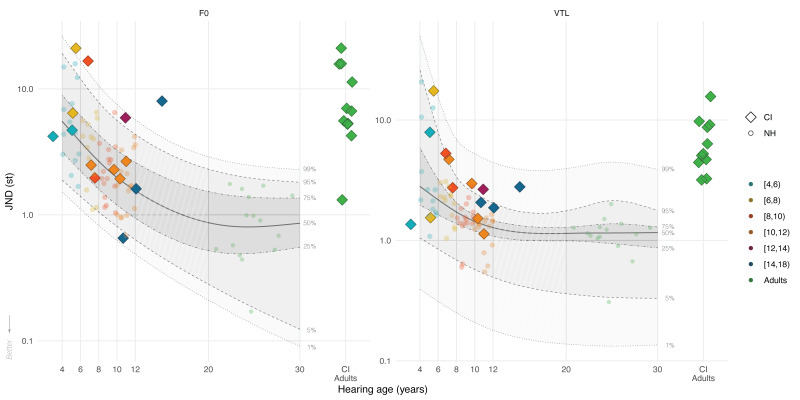


**Figure:**
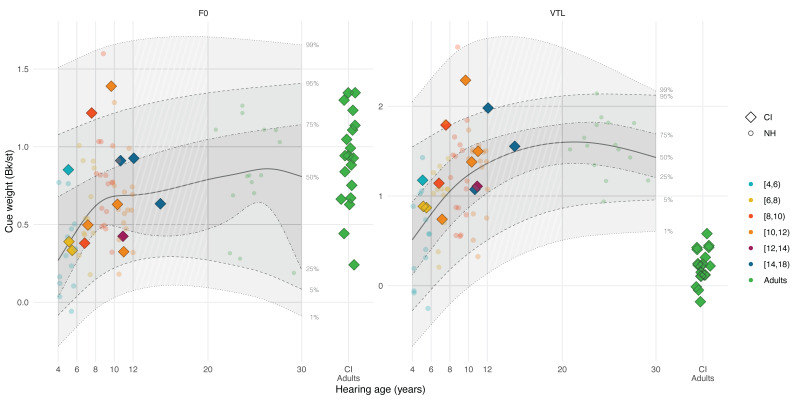


**Figure:**
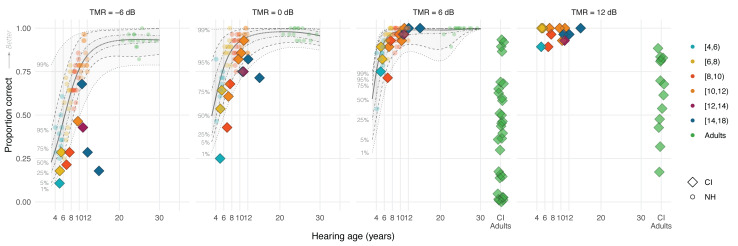


**Figure:**