# Supplementary figures and images for: Prelingually Deaf Children With Cochlear Implants Show Better Perception of Voice Cues and Speech in Competing Speech Than Postlingually Deaf Adults With Cochlear Implants
Source: Ear Hear. 2024 Apr 15;45(4):952–68. doi: 10.1097/AUD.0000000000001489 (PMC11175806; doi:10.1097/AUD.0000000000001489)

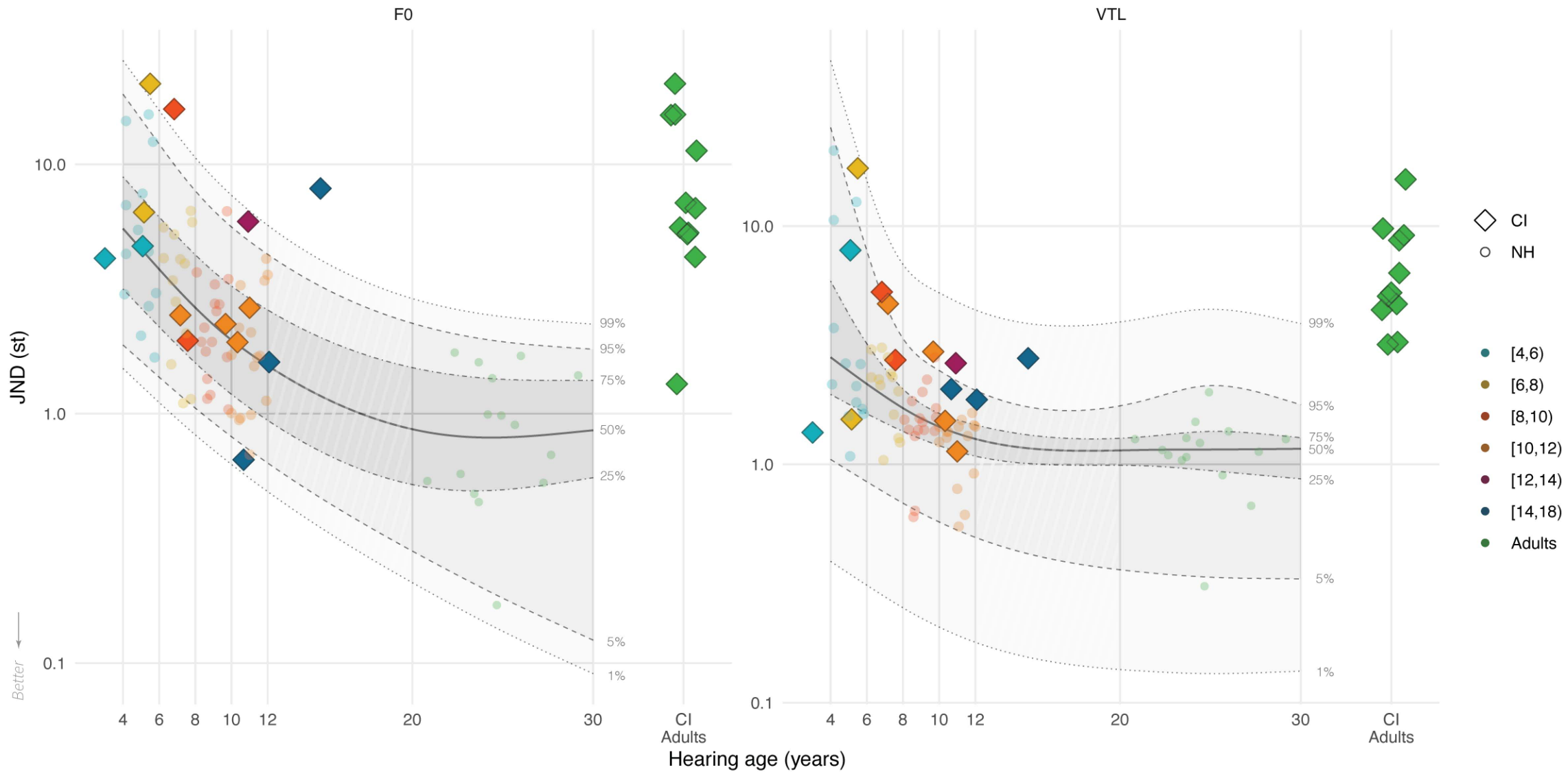

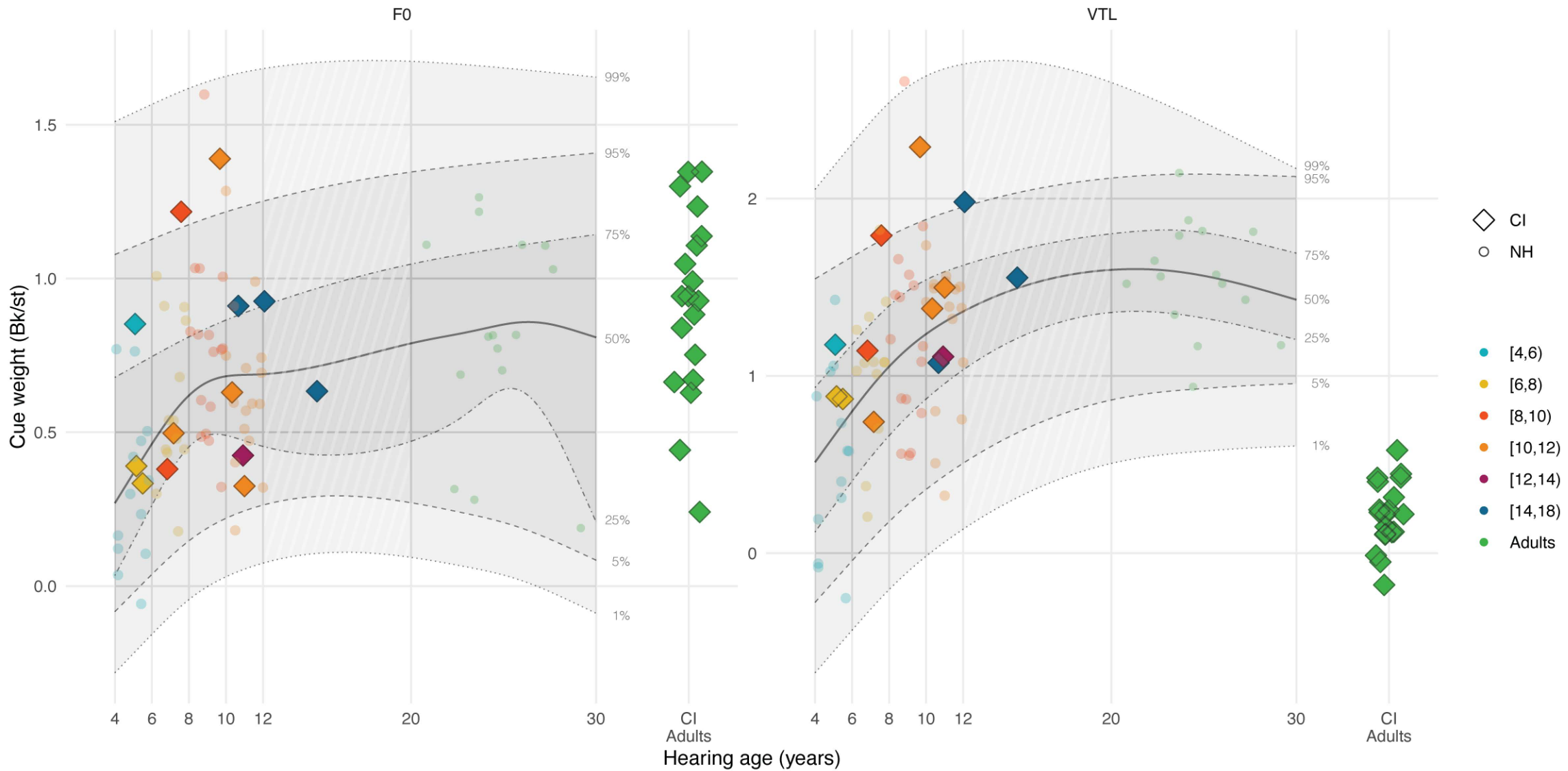

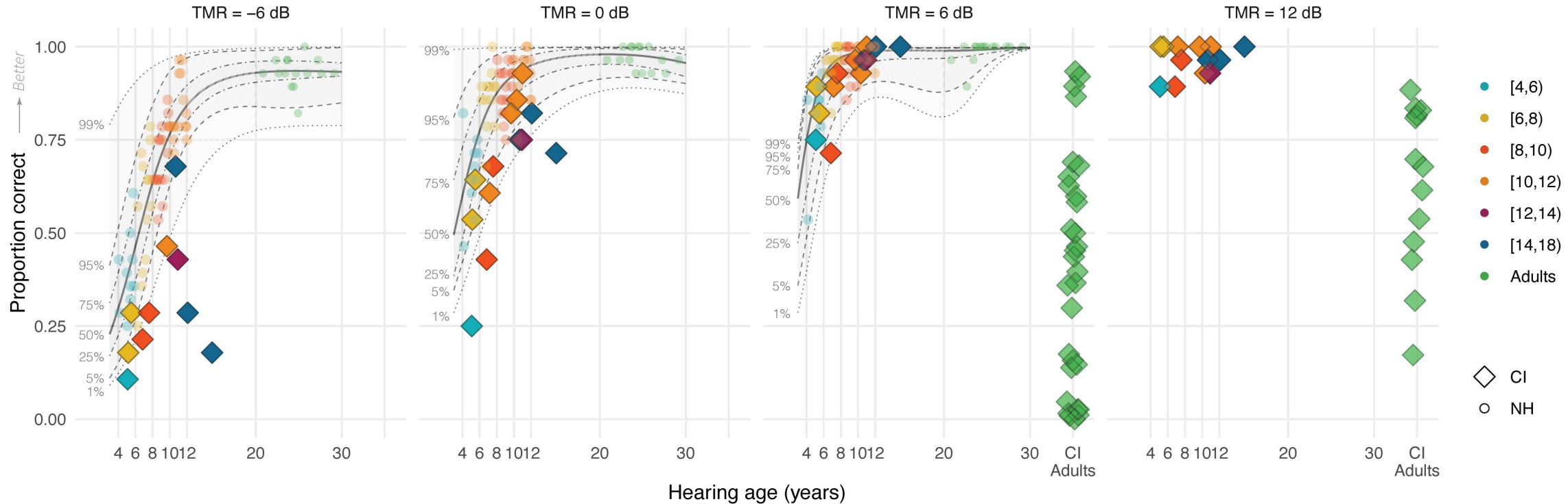

F0

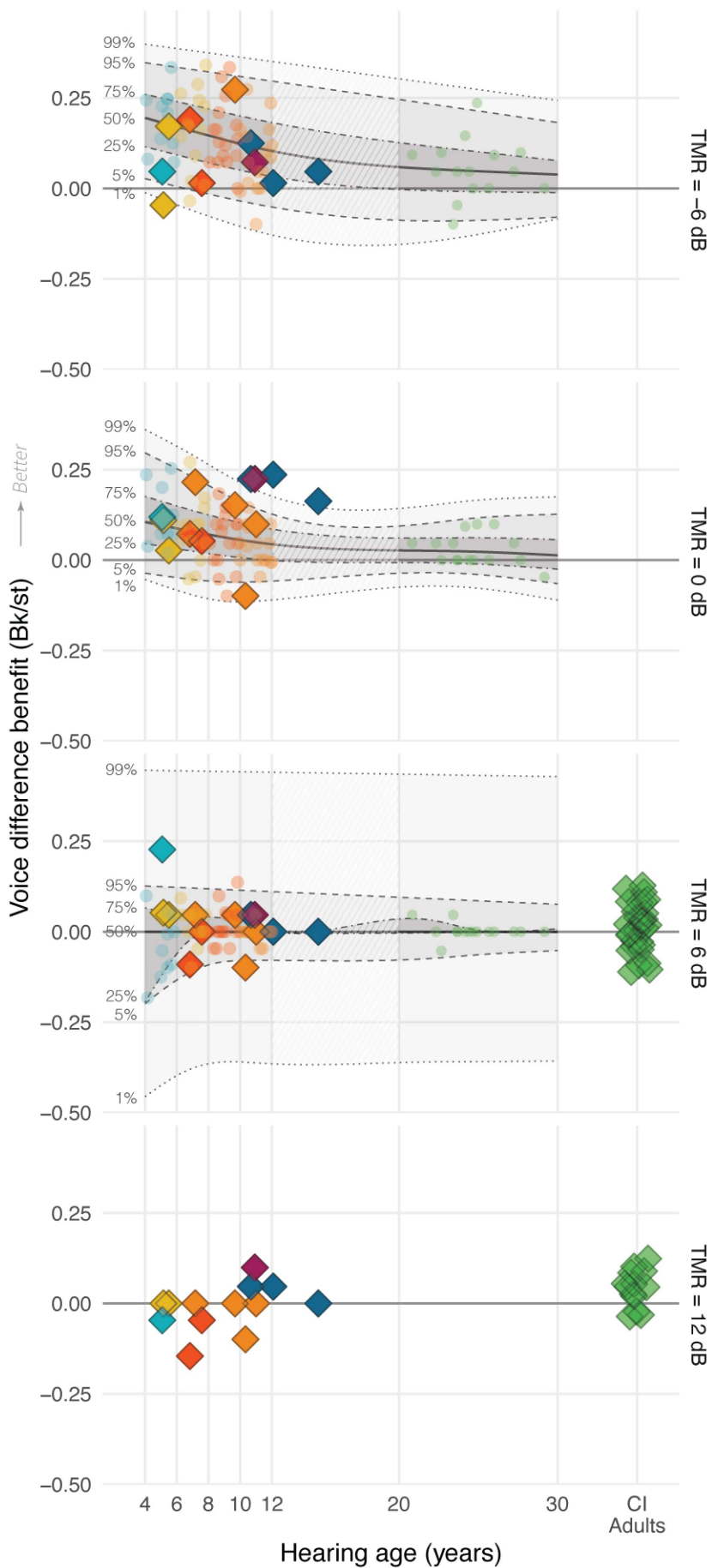

VTL

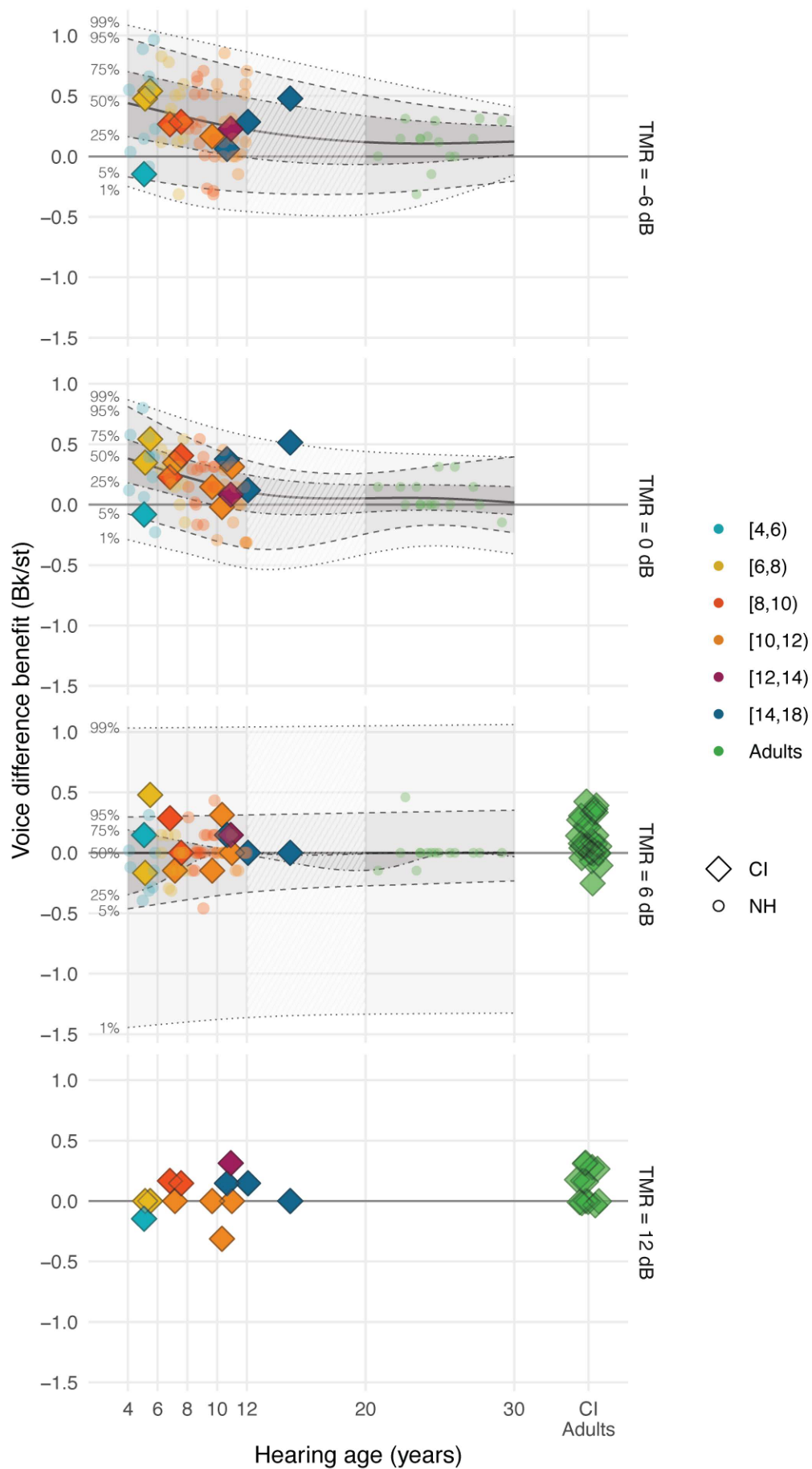

Supplement: Supplementary file 1 [file aud-45-0952-s001.pdf]
